# The impact of the termination of Lymphatic Filariasis mass drug administration on Soil-transmitted Helminth prevalence in school children in Malawi

**DOI:** 10.1371/journal.pntd.0012639

**Published:** 2026-02-25

**Authors:** Faduma Farah, Claudio Fronterrè, Mark J. Taylor, Armelle Forrer

**Affiliations:** 1 Department of Tropical Disease Biology, Liverpool School of Tropical Medicine, Liverpool, United Kingdom; 2 Department of Applied Health Sciences, University of Birmingham, Birmingham, United Kingdom; 3 Department of Clinical Sciences, Liverpool School of Tropical Medicine, Liverpool, United Kingdom; Universidad de la República Uruguay: Universidad de la Republica Uruguay, URUGUAY

## Abstract

**Background:**

Soil-transmitted helminths (STH) have been passively targeted through the implementation of mass drug administration (MDA), with the drugs ivermectin and albendazole, against the parasitic disease, lymphatic filariasis (LF). In Malawi, LF MDA was administered to communities between 2008 and 2014. The aim of this analysis was to estimate the impact of LF MDA and its termination on STH prevalence in school aged children (SAC).

**Methodology:**

School survey data of STH prevalence in Malawi were obtained through the ESPEN website. The surveys spanned the periods before (1998–2004), during (2012–2014) and after LF MDA (2015–2019). Bayesian mixed-effects models were fitted to estimate the impact of LF MDA termination, and other STH risk factors, on the odds of infection, as well as generate predictions of nationwide STH prevalence during and after LF MDA.

**Principal findings:**

SAC after the termination of LF MDA had 1.8 times greater odds of *A. lumbricoides* infection compared to SAC during the implementation of LF MDA (95% credible interval (CI): 1.03 – 3.35), despite ongoing STH preventive chemotherapy (PC) targeting SAC. Predictions indicate majority of districts increased in their probability of exceeding 2% *A. lumbricoides* prevalence in SAC after the termination of LF MDA, with Chitipa, Mulanje and Nsanje districts estimated to have > 80% probability of exceeding 2% prevalence.

**Conclusions/significance:**

An overall resurgence in *A. lumbricoides* infections after LF MDA was estimated in SAC, despite ongoing annual STH PC. This suggests STH PC could not sustain the prevalence levels achieved in SAC under community-wide LF MDA. The potential role of drug resistance in this resurgence calls for urgent investigation. Understanding how this resurgence corresponds to prevalence of moderate and heavy infections should be a priority for future research.

## Author summary

Soil-transmitted helminths (STH) are a group of parasitic worms that cause infections. They cause significant morbidity in children and women and the World Health Organisation (WHO) recommends the mass distribution of drugs to these populations as preventive chemotherapy (PC). In Malawi, distribution of albendazole to school age children (SAC) against STH has been conducted annually since at least 2012, as STH PC. In the past, Malawi also conducted mass drug administration (MDA) with albendazole and ivermectin to whole communities against another parasitic disease, lymphatic filariasis (LF). The aim of this study was to investigate the impact of terminating LF MDA on STH prevalence in SAC. We found that after the end of the programme, SAC had around 1.8 times the odds of infection for one species of STH, compared to SAC during the time of LF MDA. This indicates a resurgence in infection, despite ongoing STH PC, after the end of LF MDA and suggests STH PC could not sustain prevalence levels achieved under LF MDA. To better understand the lack of community-wide distribution and its impact, monitoring of STH prevalence and infection intensity with more sensitive diagnostics needs to be prioritised to avoid further resurgence of infection.

## Introduction

Soil-transmitted helminths (STH) are parasitic worm infections that predominately burden low and middle income (LMIC) tropical countries in areas with limited access to adequate sanitation. They are classed as a neglected tropical disease (NTD) and there are an estimated 1.5 billion cases globally [1]. In 2019, the STH burden was estimated to be equivalent to 1.9 million disability-adjusted life years [2]. The main STH species are *Ascaris lumbricoides*, *Trichuris trichiura* and the hookworms *Ancylostoma duodenale* and *Necator americanus*. Infection occurs through either ingestion of the helminth eggs (*A. lumbricoides* and *T. trichura*) or direct skin penetration of larvae (hookworms), which are both acquired from faecal contaminated soil. Chronic infections can lead to malnutrition, iron-deficiency anaemia and stunting of child development, making women of reproductive age and children at significant risk of morbidity [3,4]. The prevalence and infection intensity of *A. lumbricoides* and *T. trichiura* are highest in children aged 5–15 years [3]. In contrast, hookworm infection levels increase progressively with age and peak during adulthood [5].

The World Health Organisation (WHO) has targeted STH for elimination as a public health problem, which is defined as < 2% prevalence of moderate and heavy intensity (M&HI) infections [6]. Preventive chemotherapy (PC) programmes with the anthelmintic drugs, albendazole or mebendazole, are aimed at countries where prevalence exceeds 20%. STH PC is targeted primarily at school aged children (SAC) through school distributions. Pre-school aged children and women of reproductive age are also target groups for PC. However, coverage of pre-school aged children is highly variable and only a few countries are known to actively reach women of reproductive age [7,8]. Re-infection rates between PC rounds are high where adequate water and sanitation infrastructure is not in place [9], which threatens the attainability of the WHO goals and questions the current method of targeted deworming that is employed.

An alternative to targeted deworming of SAC is the targeting of whole communities at risk. Modelling studies have shown that elimination of STH or achievement of the WHO goals need community-wide mass drug administration (MDA) [10,11]. As a result, trials are currently underway to determine the ability of community-wide MDA to interrupt transmission of STH [12]. An example of community-based MDA that passively targets STH infections is MDA to eliminate the parasitic disease lymphatic filariasis (LF). This involves the distribution of albendazole in combination with either ivermectin, in countries that are also endemic for onchocerciasis, or diethylcarbamazine for a minimum of 5 years. With LF MDA currently implemented in 45 countries and 18 having completed the required number of rounds, effort is being made to understand how to sustain the impact of this on STH prevalence [13–16]. In Malawi, STH PC has been implemented nationwide since at least 2012 [17]. LF MDA was implemented in Malawi, alongside STH PC, from 2008 to 2014, when it reached its objectives and was terminated. Following successful transmission assessment surveys (TAS), the country was certified by the WHO as having eliminated LF as a public health problem in 2020 [18].

The aims of this study were to estimate the impact of stopping community-wide LF MDA on STH prevalence in SAC using data from the Expanded Special Project for Elimination of Neglected Tropical Diseases (ESPEN), determine risk factors for STH infection and predict areas of persistent STH transmission after LF MDA despite several years of community-based albendazole and ivermectin distribution and ongoing STH PC in Malawi.

## Methods

### Study area

**Fig 1:**
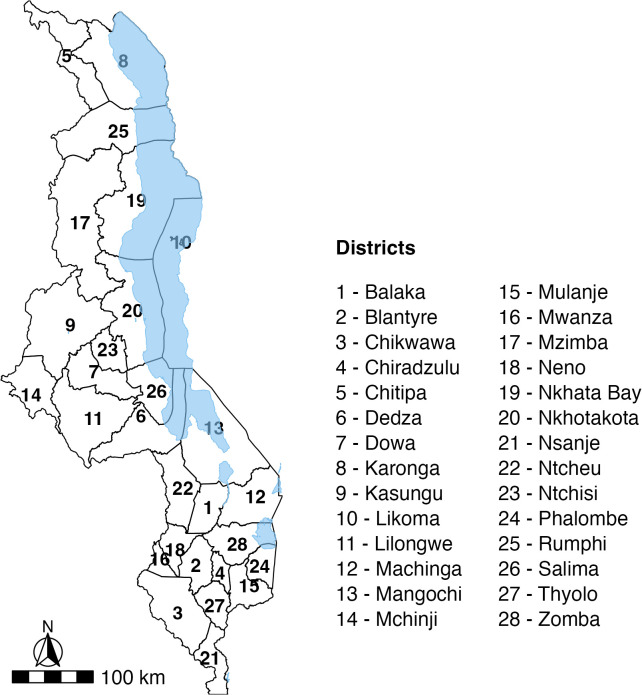
Map of Malawi with district names. Base map from GADM: https://gadm.org/download_country.html.

Malawi is a landlocked country in Sub-Saharan Africa, bordered by Mozambique, Tanzania and Zambia. Malawi has a sub-tropical climate with the rainy season between November and April. The country contains varying altitude, with mountainous areas as well as low lying regions in the south of around 90m above sea level. Malawi’s population was estimated at 20.5 million in 2022, of which 43% are children aged 0–14 years. Around 70% of the population live under $2.15 a day and 82% live in rural areas [19]. A map of Malawi with district boundaries is presented in Fig 1.

The intestinal parasitic infections that are endemic to Malawi include STH and *Schistosoma mansoni* [20]. STH PC is delivered to SAC once annually through school distributions, using the drug albendazole. This has been ongoing since at least 2012 nationwide [18]. Other control strategies of relevance to this study include LF MDA and MDA against onchocerciasis. LF MDA was conducted annually from 2008 to 2014 in Malawi, with the drug combination of albendazole and ivermectin, which was distributed within the community to those above the age of five [21]. Onchocerciasis MDA has been ongoing since 2000 in eight southern endemic districts and involves the community distribution of ivermectin to those above five years of age.

### Data sources

*A. lumbricoides*, *T. trichiura* and hookworm prevalence, obtained from surveys conducted in schools from 1998 to 2018 in Malawi, and coverage of STH PC by district from 2016 to 2019 was downloaded from ESPEN, which collates data on NTDs in African countries to summarise progress to elimination and is openly available [22]. Majority of the prevalence surveys were as part of the Ministry of Health STH monitoring activities, as recommended by the WHO [23]. The STH prevalence data from school surveys were also obtained directly from the Malawi Ministry of Health STH & Schistosomiasis control programme. Data on LF MDA coverage and the number of rounds completed by district was obtained from the Malawi Ministry of Health LF control programme. Districts endemic for onchocerciasis were identified from ESPEN

Environmental, demographic and water, sanitation and hygiene (WASH) variables that are known to potentially be associated with STH prevalence [24,25] were downloaded from open sources. Data sources with their spatial and temporal resolution, as well as maps of the environmental variables across Malawi are available in S1 Appendix and S1 Fig respectively.

### Exploratory analysis and variable selection

R software version 4.2.2 was used to combine and clean STH prevalence data as well as derive variables for assessment as risk factors for STH infection. This included environmental, demographic and WASH variables as well as time period of LF MDA (before LF MDA, during LF MDA and after LF MDA), LF MDA coverage by district, LF MDA number of completed rounds by district, STH coverage by district from 2016 to 2019 and districts classed as endemic for onchocerciasis. All mapping was done on R or QGIS version 3.16 [26]. STH prevalence data obtained from ESPEN and directly from the Ministry of Health were combined and assessed for any differences.

*A. lumbricoides*, *T. trichiura* and hookworm prevalence were assessed separately. Instances where non-zero prevalence comprised less than 5% of the total dataset were excluded from further analysis due to data sparsity. On identical grounds, exclusions were made if the proportion of data available in each time period, before, during or after LF MDA, was < 5% for each parasite. Missing STH PC coverage was imputed using random regression imputation, which aimed to predict the missing coverage levels by district and year using regression modelling in two stages. Details are provided in S1 Appendix. Histogram of the imputed STH PC coverage alongside a histogram of the available STH PC coverage is available in S2 Fig. Annual summaries were created for the environmental variables where applicable, which included the yearly average, median, minimum and maximum, for use in variable selection. Non-linear relationships between continuous variables and the observed prevalence were identified using plots of the variables against the empirical logit transformation of the prevalence [27] and all continuous variables were standardised.

As the first step in variable selection, univariate analyses with a logistic regression model were conducted to identify the best type of summary measure, as described previously, of relevant environmental variables, based on the Akaike Information Criterion (AIC) of the resulting model. The AIC was used as a selection criterion with an aim to improve model predictive performance as it serves as an approximation of the out-of-sample predictive error [28,29]. Presence of Pearson’s correlation coefficients exceeding 0.7 between continuous variables were examined as well as associations between categorical variables using the Chi-squared test of independence. A selection was made between dependent or correlated variables based on the AIC of its respective univariate model. Stepwise variable selection, using both forwards and backwards elimination, based on the AIC was then conducted to form the final list of variables to be included in the multivariate model for risk factor assessment and predictions of prevalence during and post-LF MDA.

### Variogram for assessing spatial correlation

Variograms to assess the presence of residual spatial correlation in the data were created using a Bayesian mixed-effects logistic regression model for each parasite with their respective final list of variables, fitted with the rstanarm package [30]. The presence of spatial correlation is indicated if the computed variogram falls outside of the 95% confidence interval limits [31]. Details on the formation of the variograms are available in S1 Appendix.

### Non-spatial Bayesian logistic regression model

In the case of no evidence for spatial correlation, the following mixed-effects model was fitted for each parasite for risk factor assessment and nationwide predictions of prevalence. Weakly informative prior distributions, a normal distribution with mean 0 and variance 2.5, were used for the intercept and the coefficients.


$$\text{log}\left(\frac{P\left(x_{ij}\right)}{\text{1}-P\left(x_{ij}\right)}\right)=\alpha_{\text{0}}+u_{i}+v_{j}+d{\left(x_{ij}\right)}^{T}\beta$$

$$u_{i}\;\sim \;N\left(\text{0},\;\sigma_{u}^{\text{2}}\right)$$

$$v_{j}\;\sim \;N\left(\text{0},\;\sigma_{v}^{\text{2}}\right)$$

Here the log-odds of infection prevalence at school $x_{ij}$ is equal to the global intercept  (α plus random effects at school-level $\left(u_{i}\right),\;$district-level $(v_{j}\backslash )$ and $d{\left(x_{ij}\right)}^{T}\beta$, the vector of explanatory variables at school $x_{ij}$ with β as their coefficients, which represent the fixed effects. Each random effect is a set of independent zero-mean Gaussian variables with variances $\sigma_{u}^{\text{2}}$ and $\sigma_{v}^{\text{2}},$ respectively. Markov chain Monte Carlo (MCMC) simulations with a burn-in of 1,000 followed by 2,000 iterations per chain, were run over four chains. A total of 4,000 simulations were generated, of which model estimates and their posterior distributions were retrieved. Model coefficients were converted to odds ratios, which are presented alongside 95% credible intervals. To assess the overall nationwide impact of terminating LF MDA, the odds of infection in SAC in the time period after LF MDA, compared to during LF MDA implementation, were obtained.

To assess how much of the variability between schools or districts was explained by the explanatory variables used in the model, the relative decrease in school-level variance and district-level variance was computed, details on this calculation are provided in S1 Appendix. The fit of each final model was assessed through posterior predictive checks [32], where the standard deviation, maximum prevalence and the proportion of zero prevalence schools observed in the data were compared to those predicted by the model. This highlights any limitations in the model predictions and cases where the model fails to correspond to the observed data if it falls outside of the range of the predicted distribution. Further details are available in S1 Appendix.

Predictions of prevalence across Malawi for two time periods, during the implementation of LF MDA and after the termination of LF MDA, were produced from each model on a 2 km^2^ grid alongside their 95% predictive intervals. Maps displaying the probability of exceeding either 2% or 20% prevalence during and after LF MDA (“exceedance probability”) were generated from these predictions, as well as the change in the exceedance probability after LF MDA. The thresholds of 2% and 20% prevalence reflect WHO thresholds of assessing progress of STH PC after five years, where PC can be stopped if the region achieves < 2% prevalence in the target population while PC is continued at the same frequency if prevalence is ≥ 20% [6]. Areas of persistent transmission were defined here as areas where the probability of exceeding 2% prevalence of either parasite was > 80% after LF MDA. To assess the spread or uncertainty in the predictions while excluding extreme values, the relative interquartile range (IQR) ($\frac{IQR}{median}$) in areas where the median predicted prevalence was above 0%, was calculated and mapped.

### Sensitivity analysis

As additional districts were surveyed after LF MDA, the non-spatial mixed-effects Bayesian logistic regression models on each parasite were repeated with only the districts surveyed both during and after LF MDA. This was to identify whether the impact of terminating LF MDA on the odds of infection were maintained. Random effects at school-level and district-level were kept as well as the final set of explanatory variables selected for each model.

## Results

### STH prevalence before, during and after LF MDA in Malawi

Two differences were identified between the data sources for STH prevalence. Data from the Ministry of Health contained school surveys from the year 2019, which was not available on ESPEN at the time of this analysis. The second difference was that the ESPEN data had an additional nine schools surveyed in 2017 that were not apparent in the data from the Ministry of Health. The remaining data were identical between the two sources. Therefore, the ESPEN data was taken forward for analysis with the addition of school surveys from 2019, acquired from the Ministry of Health data.

LF MDA coverage in Malawi achieved above 65% coverage for over five years, fulfilling the target set for LF MDA. It was implemented between 2008 and 2014 and coverage data available at district level indicated that average nationwide coverage of LF MDA ranged from 78% to 84% during the seven years of implementation (S3 Fig). Two districts were classed as non-endemic for LF, Chitipa and Likoma Island. Despite that, Chitipa received one round of LF MDA in 2014 at 85% coverage and Likoma received two rounds, in 2010 and 2014, at 78% and 90% coverage respectively.

**Fig 2:**
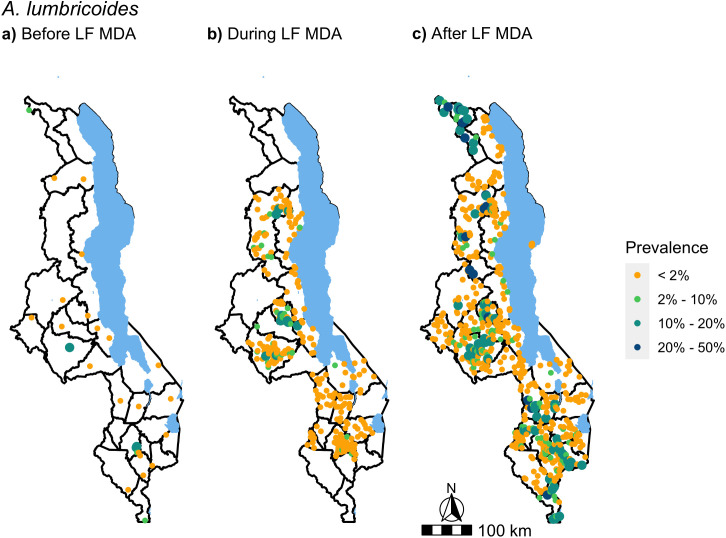
*A. lumbricoides* prevalence in SAC during the three phases of LF MDA, before (a), during (b) and after (c). Data were obtained from ESPEN and the National Schistosomiasis & STH control programme in Malawi. Survey years range from 1998 to 2019. 33 schools were surveyed before LF MDA (1998 – 2004), 337 schools during LF MDA (2012 – 2014) and 657 after LF MDA (2015 – 2019). Base map from GADM: https://gadm.org/download_country.html.

**Fig 3:**
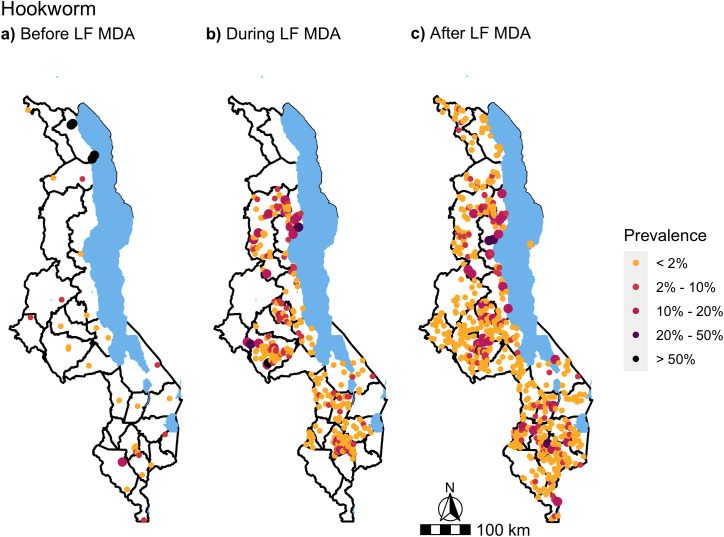
Hookworm prevalence in SAC during the three phases of LF MDA, before (a), during (b) and after (c). Data was obtained from ESPEN and the National Schistosomiasis & STH control programme in Malawi. Survey years range from 1998 to 2019. 33 schools were surveyed before LF MDA (1998 – 2004), 337 schools during LF MDA (2012 – 2014) and 657 after LF MDA (2015 – 2019). Base map from GADM: https://gadm.org/download_country.html.

Of 1076 schools surveyed between 1998 and 2019 across the 28 districts of Malawi, the final dataset in this analysis included 1027 schools. Coordinates were not available or incorrect for a total of nine schools and at 40 school locations, data on WASH variables were not available. A median number of 30 SAC were surveyed in each school (range: 3 – 280), of which four schools examined fewer than 11 children. The total number of SAC examined in this dataset was 34,790. Figs 2 and 3 show the location and number of schools surveyed across the three LF MDA time periods. The first period, before LF MDA (Figs 2a and 3a), comprised of surveys conducted from 1998 to 2004. The second period, during LF MDA (Figs 2b and 3b), included surveys conducted from 2012 to 2014 and the third period, after LF MDA, (Figs 2c and 3c), included surveys conducted from 2015 to 2019. The majority of the data, 64%, was collected after LF MDA (Figs 2c and 3c). Before LF MDA, estimated prevalence was only available from 33 schools nationwide (Figs 2a and 3a).

*Trichuris trichiura* was the least prevalent of the STH in SAC across the three time periods of LF MDA. The average *T. trichiura* prevalence before LF MDA in the 33 schools was 0.2%, with a range in the school-level prevalence of 0% to 1.8%. A greater number of schools were surveyed per district during and after LF MDA, allowing for summary at the district level. During and after LF MDA, the average *T. trichiura* prevalence at district level ranged from 0% to 0.7% and 0% to 1.3% respectively. Only 4% of schools (36/994) assessed during and after LF MDA had *T. trichiura* prevalence above 0%.

**Fig 4:**
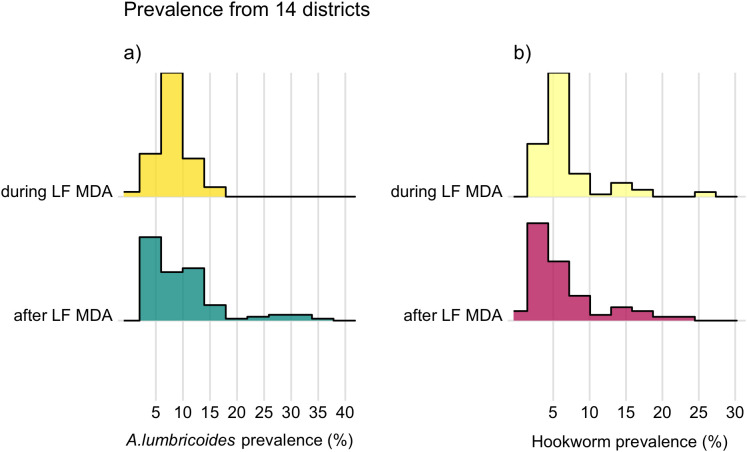
The distribution of the above 0% school-level prevalence in SAC during and after LF MDA in Malawi*. A. lumbricoides* (a) and hookworm (b). Data was obtained from ESPEN and the National Schistosomiasis & STH control programme in Malawi. *A. lumbricoides* and hookworm prevalence from 14 districts surveyed both during and after LF MDA are presented, which include 337 schools surveyed during LF MDA and 410 schools surveyed after LF MDA.

Estimated *A. lumbricoides* prevalence at each school, before, during and after LF MDA are presented in Fig 2. Before LF MDA, the average *A. lumbricoides* prevalence, out of 28 schools that were examined for this parasite, was 2.5% with a range in the school-level prevalence of 0% to 15.4%. When analysed at the district level, 93% of districts (13/14) during LF MDA had below 2% average *A. lumbricoides* prevalence, with Ntchisi district just above this threshold at 4%. After LF MDA, the percentage of districts with an average below 2% prevalence declined to 64% (18/28). An increase in the range of school-level *A. lumbricoides* prevalence was observed from 15.9% during LF MDA to 33.3% after LF MDA. As not all districts were surveyed in both time periods, during and after LF MDA, Fig 4 presents the distribution of school-level prevalence of *A. lumbricoides* and hookworm above 0%, to emphasise clearly the change in the range of prevalence observed, in the 14 districts with data across both time periods.This showed that the increase in the range of *A. lumbricoides* prevalence observed, remained when the data was restricted to those districts (Fig 4a).

Higher levels of hookworm prevalence were observed before and during LF MDA when compared to *A. lumbricoides* (Fig 3). Before LF MDA, the school-level prevalence ranged from 0% to 77.3% across the 33 schools surveyed, with an average of 9.8%. During LF MDA, the average hookworm prevalence at district level ranged to a high of 6.5% in Nkhata Bay district. In addition, the percentage of districts that had less than 2% average hookworm prevalence was 71% (10/14) during LF MDA, which increased after LF MDA to 75% (21/28 districts). The range of school-level hookworm prevalence also decreased from 25% during LF MDA to 22.5% after LF MDA and this decrease was maintained in the 14 districts that were assessed in both time periods (Fig 4b).

### Risk factors of *A. lumbricoides* and hookworm infection

Due to the sparsity of data before LF MDA and on *T. trichiura* prevalence, these points were excluded from further modelling. The four schools that examined fewer than 11 children were also removed from further analysis.

**Fig 5:**
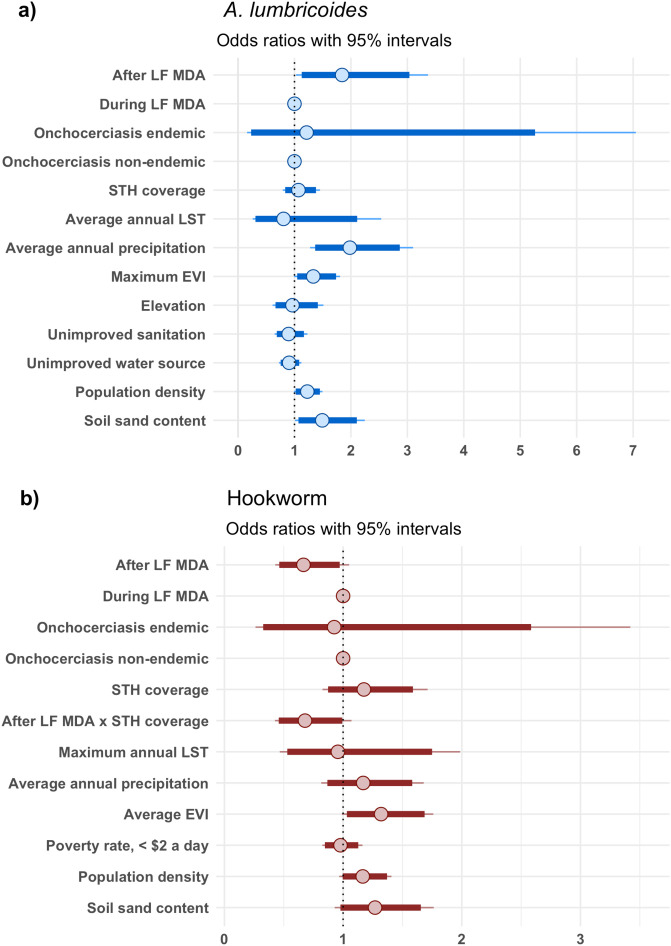
Risk factors of *A. lumbricoides* (a) and hookworm (b) prevalence in SAC in Malawi. Obtained from a multivariate mixed-effects Bayesian logistic regression model of 990 schools surveyed from the years 2012 to 2019, with a total sample of 32,665 SAC.

The data showed a lack of spatial correlation in either parasite as assessed from the variograms, which are able to viewed in S4 Fig. Therefore, non-spatial mixed-effects Bayesian logistic regression models, accounting for school level and district level heterogeneity, were fit on *A. lumbricoides* and hookworm prevalence in SAC, across the two time periods, during and after LF MDA. The results are presented in Fig 5. SAC after the termination of LF MDA were estimated to have 1.8 times the odds of *A. lumbricoides* infection compared to SAC during the implementation of LF MDA (Odds Ratio (OR): 1.8, 95% credible interval (CI): 1.03 – 3.35), which indicated a resurgence in prevalence after LF MDA. SAC in areas of increased precipitation and areas of increased soil sand content were associated with greater odds of *A. lumbricoides* infection at ratios of 2.0 (95% CI: 1.28 – 3.10) and 1.5 (95% CI: 1.01 – 2.25), respectively*.* Borderline significant associations with increased odds of *A. lumbricoides* infection were also found with greater population density (OR: 1.2, 95% CI: 0.99 – 1.50) and higher levels of EVI (OR: 1.3, 95% CI: 0.99 – 1.76).

In contrast to the *A. lumbricoides* model, SAC after LF MDA were estimated to have reduced odds of hookworm infection compared to SAC during LF MDA (OR: 0.7, 95% CI: 0.43 – 1.05). However, this estimate slightly surpassed the threshold for statistical significance. Similarly, there were no statistical significant associations for the remainder of the variables in the hookworm model. Although, the effect where SAC in districts with increased STH PC coverage were estimated to have reduced odds of infection after LF MDA but not during LF MDA implementation, as indicated by the interaction term (OR: 0.7, 95% CI: 0.43 – 1.07), was just outside the margins of statistical significance.

The inclusion of the explanatory variables used in the hookworm model, explained 13% of the variability in hookworm prevalence between districts. With the *A. lumbricoides* model, the explanatory variables explained 6% of the variability in *A. lumbricoides* prevalence between schools (S1 Table). The assessment of model fit showed a tendency of both the *A. lumbricoides* and hookworm models to predict a greater standard deviation in the school-level prevalence, so greater variability, than what was observed in the data (S7 Fig). However, in both cases, the observed standard deviation was still within the range of the predicted standard deviation distribution, indicating the model did not fail to correspond to the observed data (S7 Fig). Likewise, the maximum prevalence and proportion of zero prevalence’s in the observed data were within the range predicted by the model for both *A. lumbricoides* and hookworm (S7 Fig).

### Predictions of *A. lumbricoides* and hookworm prevalence in SAC during and after LF MDA

**Fig 6:**
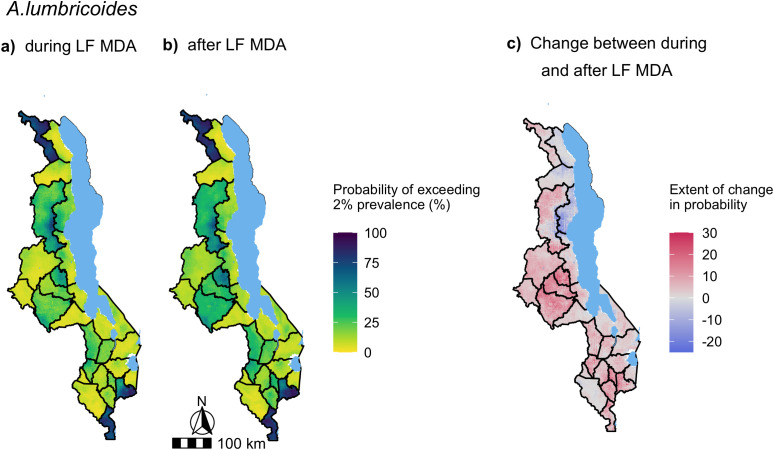
Maps of the probability of exceeding 2% predicted *A. lumbricoides* prevalence in Malawi during (a) and after LF MDA (b). The change in the probability of exceeding 2% *A. lumbricoides* prevalence was calculated from the period during LF MDA to after LF MDA, where areas highlighted in red increased in their probability of exceeding 2% prevalence after LF MDA was stopped and areas in blue decreased, with darker shades indicating a greater increase/decrease in percentage points **(c)**. Predictions were generated from a multivariate mixed-effects Bayesian logistic regression model, presented in Fig 5, of 990 schools surveyed from the years of 2012 to 2019, with a total sample of 32,665 SAC. Base map from GADM: https://gadm.org/download_country.html.

**Fig 7:**
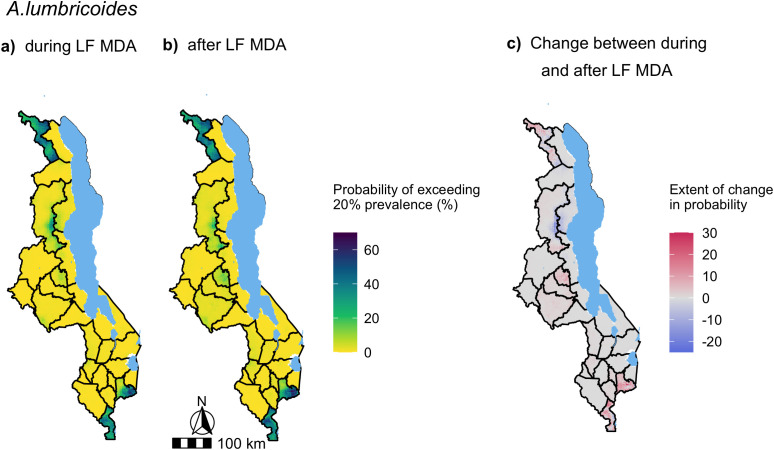
Maps of the probability of exceeding 20% predicted *A. lumbricoides* prevalence in Malawi during (a) and after LF MDA (b). The change in the probability of exceeding 20% *A. lumbricoides* prevalence was calculated from the period during LF MDA to after LF MDA, where areas highlighted in red increased in their probability of exceeding 20% prevalence after LF MDA was stopped and areas in blue decreased, with darker shades indicating a greater increase/decrease in percentage points **(c)**. Predictions were generated from a multivariate mixed-effects Bayesian logistic regression model, presented in Fig 5, of 990 schools surveyed from the years of 2012 to 2019, with a total sample of 32,665 SAC. Base map from GADM: https://gadm.org/download_country.html.

Nationwide predictions of *A. lumbricoides* and hookworm prevalence in SAC during and after LF MDA were generated from the full multivariate mixed-effects models presented in Fig 5. The predicted median of *A. lumbricoides* and hookworm prevalence across the country at 2 km resolution and their uncertainty are presented in S5 and S6 Figs respectively. To illustrate the impact of LF MDA on STH distribution and abundance, the change in probability of *A. lumbricoides* and hookworm prevalence exceeding 2% (Fig 6c) and 20% (Fig 7c) after LF MDA are presented.

Most of the country exhibited an increased probability of exceeding 2% *A. lumbricoides* prevalence after LF MDA with the highest rise predicted in central and southern regions (Fig 6c). The increase was over 10 percentage points in the central districts of Lilongwe, Dowa and Ntchisi, and the southern districts of Mulanje and Chiradzulu (Fig 6c). Only the region stretching from Nkhata Bay, through Rumphi to Karonga district was predicted to have a decreased probability of exceeding 2%. The change in the probability of *A. lumbricoides* prevalence exceeding 20% in SAC after LF MDA was much less significant with the majority of the country predicted to experience within ±10 percentage points change in their probability (Fig 7c).

Most importantly, areas of persistent transmission, defined as those with a probability of exceeding 2% *A. lumbricoides* prevalence greater than 80% after LF MDA, were predicted in southern and central areas in Chitipa district, north-western areas in Nsanje district and the mountainous zone of Mulanje district (Fig 6b). No areas were predicted to have > 80% probability of exceeding 20% *A. lumbricoides* prevalence after LF MDA (Fig 7b).

**Fig 8:**
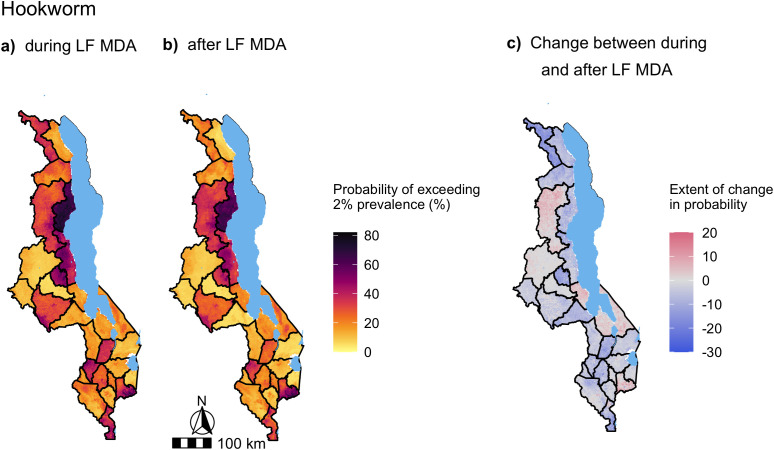
Maps of the probability of exceeding 2% predicted hookworm prevalence in Malawi during (a) and after LF MDA (b). The change in the probability of exceeding 2% hookworm prevalence was calculated from the period during LF MDA to after LF MDA, where areas highlighted in red increased in their probability of exceeding 2% prevalence after LF MDA was stopped and areas in blue decreased, with darker shades indicating a greater increase/decrease in percentage points **(c)**. Predictions were generated from a multivariate mixed-effects Bayesian logistic regression model, presented in Fig 5, of 990 schools surveyed from the years of 2012 to 2019, with a total sample of 32,665 SAC. Base map from GADM: https://gadm.org/download_country.html.

**Fig 9:**
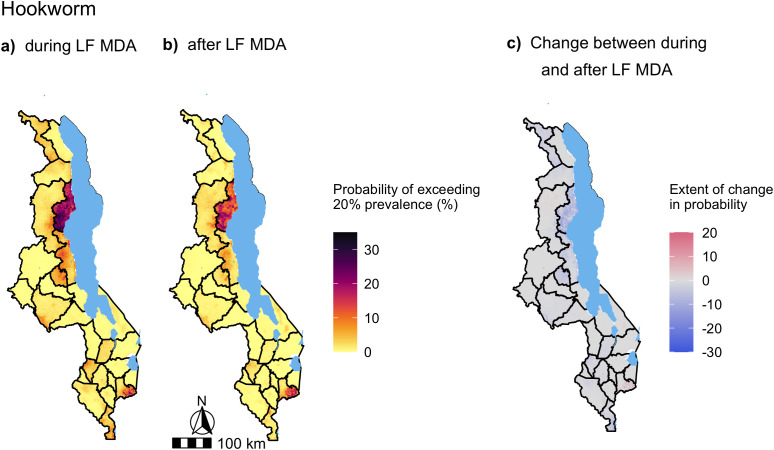
Maps of the probability of exceeding 20% predicted hookworm prevalence in Malawi during (a) and after LF MDA (b). The change in the probability of exceeding 20% hookworm prevalence was calculated from the period during LF MDA to after LF MDA, where areas highlighted in red increased in their probability of exceeding 20% prevalence after LF MDA was stopped and areas in blue decreased, with darker shades indicating a greater increase/decrease in percentage points **(c)**. Predictions were generated from a multivariate mixed-effects Bayesian logistic regression model, presented in Fig 5, of 990 schools surveyed from the years of 2012 to 2019, with a total sample of 32,665 SAC. Base map from GADM: https://gadm.org/download_country.html.

In distinction to the *A. lumbricoides* prevalence predictions, the change in the probability of hookworm prevalence exceeding either 2% or 20% after LF MDA predominantly decreased (Figs 8c and 9c). Only three districts, Mangochi, Mulanje and Mzimba were predicted to increase after LF MDA (Figs 8c and 9c). No areas of persistent hookworm transmission were identified, where the probability of exceeding 2% hookworm prevalence was > 80% after LF MDA (Fig 8b).

### Sensitivity analysis

**Fig 10:**
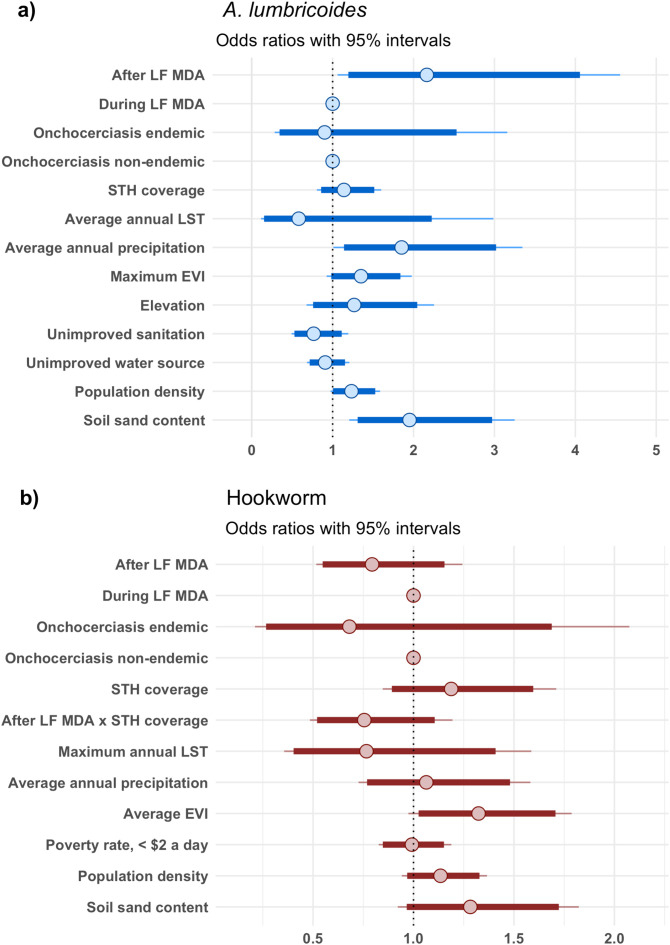
Impact of LF MDA termination on *A. lumbricoides* (a) and hookworm (b) prevalence in SAC in Malawi. Obtained from a multivariate mixed-effects Bayesian logistic regression model of 745 schools surveyed from the years of 2012 to 2019, with a total number of 25,491 SAC.

Results from the sensitivity analysis are presented in Fig 10. Restricting the models to the 14 districts surveyed in both time periods, during and after LF MDA (as shown in Figs 2b and 3b) reduced the total number of schools from 990 to 745, 336 surveyed during and 409 surveyed after LF MDA. The *A. lumbricoides* model, continued to indicate increased odds of infection in SAC post termination of LF MDA (OR: 2.2, 95% CI: 1.06 – 4.57), which confirmed the presence of a resurgence in infection. The odds of hookworm infection post LF MDA in SAC remained lower relative to SAC during LF MDA, however the estimate increased in uncertainty (OR: 0.8, 95% CI: 0.52 – 1.24) when compared to the full model presented in Fig 5 and remained statistically insignificant.

## Discussion

This analysis of STH prevalence data from Malawi reveals a resurgence in *A. lumbricoides* infections in SAC post implementation of LF MDA, despite ongoing annual school-based STH PC. The overall, country-wide odds of infection with *A. lumbricoides* were found to have almost doubled after the termination of LF MDA. This quick rise in *A. lumbricoides* prevalence after six years of community-wide distribution of albendazole and ivermectin MDA at > 75% coverage, suggests that this regimen in Malawi was not sufficient to interrupt *A. lumbricoides* transmission. This coincides with results from modelling that suggest more than 10 years of 75% community-wide coverage with albendazole is required to interrupt *A. lumbricoides* transmission [10].

The estimated increased odds of infection with *A. lumbricoides* after LF MDA also show that the prevalence levels achieved during a chemotherapy-based initiative as intensive as LF MDA, could not be sustained by school-based STH PC. Community-wide MDA is expected to decrease environmental contamination of the parasite to a greater extent than targeted PC, as adults, who are estimated to host around 30% of the *A. lumbricoides* infectious reservoir [33], and children not enrolled in school would be covered. This superiority of community-based distribution over a school-based strategy is supported by a previous systematic review and meta-analysis, which showed a significantly greater impact on *A. lumbricoides* prevalence reduction in SAC with community-wide distribution in comparison to targeted distribution to SAC [34]. There are 58 countries co-endemic for LF and STH, of which 13 countries are currently under post-LF MDA surveillance [35,36]. There is scope here to assess the contribution of LF MDA to STH control and elimination. Leveraging LF MDA infrastructure for the monitoring of STH during LF TAS, conducted after the implementation of LF MDA, has been discussed previously and guidelines on this integration have been published by the WHO [21,37]. Increased uptake of this could mitigate the lack of funding for STH surveillance activities and importantly provide valuable data necessary for modelling analyses to assess the impact of LF MDA programmes with various characteristics, such as coverage and duration of implementation, on STH.

Our predictions suggest that *A. lumbricoides* resurgence was not limited to certain regions as an increase in the probability of exceeding either 2% or 20% prevalence was found across the majority of the country, with the exception of some northern areas which were predicted to decrease in their probability after LF MDA. However, it did highlight the districts of Chitipa, Mulanje and Nsanje, which were estimated to have a high likelihood (> 80%) of exceeding the WHO threshold of 2% prevalence after LF MDA. The period after LF MDA marks five years of STH PC implementation in Malawi as the majority of the STH prevalence data after LF MDA was collected from 2017 to 2019 (96%, 634/657 schools). Therefore, our predictions suggest continued STH PC is required in these districts in accordance with WHO recommendations [6]. Chitipa was classed as non-endemic for LF and only received one round of LF MDA, which likely contributed to the levels observed in this district.

Low pre-MDA prevalence and transmission intensity could explain the lack of resurgence in northern areas but data for the pre-MDA era are limited. A survey conducted in the northern district of Karonga in 1999 across four schools found that hookworm infections dominated at an overall prevalence of 64%, compared to an *A. lumbricoides* prevalence of 0.4% [38]. However, another nationwide survey of 30 schools conducted in 2002, before MDA, found similar low prevalence rates of *A. lumbricoides* across other regions of the country [39]. Environmental factors are known to condition STH distribution, and we found increased odds of *A. lumbricoides* infection in areas with an increased fraction of sandy soil. Increased contamination of sandy soil, in comparison to other soil types, with *A. lumbricoides* has been described in previous studies [40–42]. The northern districts of Nkhata Bay and Rumphi are found to have reduced fractions of sandy soil, which could underly the lack of resurgence predicted in these areas. These districts are also either unsuitable or not available as agricultural land [43]. Agricultural practices are common across majority of Malawi and are often found associated with increased STH infection risk [44,45].

Contrary to the *A. lumbricoides* model, the odds of hookworm infection in SAC were lower after LF MDA, although not significantly. Decreased hookworm infection levels would be expected under community-wide LF MDA which would treat adults, among whom hookworm infection levels peak, in the absence of PC to this population [5]. Hookworm infections have also been shown to take longer to return to pre-treatment levels after MDA compared to *A. lumbricoides*, which has the highest re-infection rate of the STHs [9]. The predictions of hookworm prevalence suggest that among the widespread decline in the probability of exceeding either 2% or 20% hookworm prevalence, only Mzimba, Mangochi and Mulanje districts were predicted to increase. Mzimba and Mangochi were also found in regions that harboured the highest pre-LF MDA hookworm infection levels in SAC from the nationwide survey conducted in 2002 [39].

### Limitations

There were limitations to this analysis. Estimates of STH prevalence were not available in half of the districts during LF MDA. However, the increased odds of *A. lumbricoides* infection were confirmed by a sensitivity analysis conducted using only the 14 districts surveyed both during and after LF MDA. STH PC coverage data prior to 2016 was also unavailable and so values were assumed using random imputation with regression modelling. The lack of infection intensity data, limited result interpretation in terms of public health impact and prevented assessing whether the WHO target for morbidity control of < 2% prevalence of M&HI infections were met [6].

*A. lumbricoides*, and particularly hookworm prevalence post LF MDA, were likely to have been underestimated due to the decreased sensitivity of Kato Katz for low intensity infections [46], which are to be expected after years of community-wide albendazole distribution [47,48]. This issue is particularly salient for hookworm due to the additional challenge of egg frailty, which are rapidly destroyed following Kato Katz slide preparation [47,49,50]. Lastly, the environmental, WASH and MDA programme variables included in our models had low explanatory power. This lack of association between programmatic or environmental data and STH prevalence suggests that the current STH distribution is due to other factors.

There are two possible major underlying factors contributing to the distribution of *A. lumbricoides* and hookworm across Malawi. First, *A. lumbricoides* or hookworm infection risk appear to be highest in areas subject to migration, which include Nsanje, Mulanje, Mzimba and Mangochi. Migration of individuals is a key factor in the epidemiology of infectious diseases [51–53]. A model simulation has shown that as few as 2% of the population of an area, even one of low prevalence, moving to an area that has undergone community based MDA for five years, is enough to reduce the probability of eliminating both hookworm and *A. lumbricoides* in the treated area to below 50% [54]. Mzimba reported the highest net gain in internal migrants in the 2018 and economic opportunities at fisheries in Mangochi district attracts workers, including from neighbouring Mozambique where moderate to high prevalence of STH is reported [55–57]. Both Nsanje and Mulanje districts highlighted in the *A. lumbricoides* prevalence predictions after LF MDA, also border districts of Mozambique with moderate to high prevalence of STH [57].

The other possible underlying factor could be the emergence of reduced albendazole efficacy, through either genetic resistance or other mechanisms [58–62]. Widespread resistance has been established within helminth species of livestock and the same genetic polymorphisms in the β-tubulin gene, which confer resistance to benzimidazoles, have since been described in STH, including in field samples from Kenya, Panama, Haiti, Ghana, Mozambique and Brazil [63–68]. Studies have also reported cases of reduced drug efficacy against STH when compared to WHO reference values [69–72], including albendazole against *A. lumbricoides* infections [73]. Given that in Malawi, MDA against onchocerciasis, LF and STH have been conducted for more than a decade [74], both drug efficacy and the presence of mutations in the β-tubulin gene, should be investigated and monitored [62]. In particular when ivermectin treatment has also been shown to select for the same genetic polymorphisms described in the β-tubulin gene [75]. Cost effective approaches for genetic benzimidazole resistance monitoring would however be required and could be developed alongside molecular methods for high-sensitivity diagnostics which are needed in low infection intensity, post-MDA settings.

## Conclusion

The resurgence in *A. lumbricoides* prevalence in Malawian SAC despite STH PC calls for expanding drug distribution to the community in order to capitalise on progress made under LF MDA. The potential role of migration or population movement on increased *A. lumbricoides* and hookworm infection risk after LF MDA, also highlights the importance of regional coordination and cross-border efforts to effectively reduce STH transmission alongside country-specific policies. However, if resistance to benzimidazoles is playing a key part, community-wide MDA with albendazole would have limited impact on infection rebound. The potential role of benzimidazole resistance calls for urgent investigation in areas of persistent or resurging STH infection. Integration of STH monitoring into LF TAS can provide a platform for resistance monitoring, achieving cost-effectiveness through pooling of faecal samples. Alternatively, environmental surveillance could also provide a low-cost approach to address this challenge. Crucially, an understanding of how this resurgence translates to infections of M&HI and morbidity, which is the focal point of the WHO targets for STH control, should be a priority for future research.

## Supporting information

S1 Fig Distribution of the environmental variables used in the *A. lumbricoides* and hookworm models. Base map from GADM: https://gadm.org/download_country.html.
(DOCX)

S2 Fig Distribution of imputed and the available STH preventive chemotherapy coverage. (DOCX)

S3 Fig Mean coverage of Lymphatic Filariasis mass drug administration in Malawi by year. Horizontal dashed line indicates the 65% coverage target set for LF MDA.
(DOCX)

S4 Fig Variograms from the full *A. lumbricoides* and hookworm models. (DOCX)

S5 Fig *A. lumbricoides* prevalence predictions. Median of the predicted prevalence after LF MDA alongside 95% predictive intervals and the relative interquartile range (IQR), IQR/median of the predictions where the median is above 0%. Base map from GADM: https://gadm.org/download_country.html.
(DOCX)

S6 Fig Hookworm prevalence predictions. Median of the predicted prevalence after LF MDA alongside 95% predictive intervals and the relative interquartile range (IQR), IQR/median of the predictions where the median is above 0%. Base map from GADM: https://gadm.org/download_country.html.
(DOCX)

S7 Fig Histograms displaying statistical summaries of the predicted vs observed prevalence. Derived from 4,000 simulations of the predicted prevalence at each school obtained from the full multivariate mixed-effects Bayesian logistic regression models presented in Fig 5. (a-c) The standard deviation, maximum school-level prevalence and percentage of zero prevalence’s predicted by the *A. lumbricoides* model for each of the 4,000 simulations, compared to its respective observed value in the data. (d-f) The standard deviation, maximum school-level prevalence and percentage of zero prevalence’s predicted by the hookworm model for each of the 4,000 simulations, compared to its respective observed value in the data.
(DOCX)

S1 Table Inclusion of the explanatory variables. The change in the variance of the random effects in the *A. lumbricoides* and hookworm models, with no explanatory variables (empty model) vs with all explanatory variables (full model).
(DOCX)

S1 Appendix Details on the methodology. (DOCX)
