# Supplementary figures and images for: The impact of the termination of Lymphatic Filariasis mass drug administration on Soil-transmitted Helminth prevalence in school children in Malawi
Source: PLoS Negl Trop Dis. 2026 Feb 25;20(2):e0012639. doi: 10.1371/journal.pntd.0012639 (PMC12956128; doi:10.1371/journal.pntd.0012639)

S1 Fig


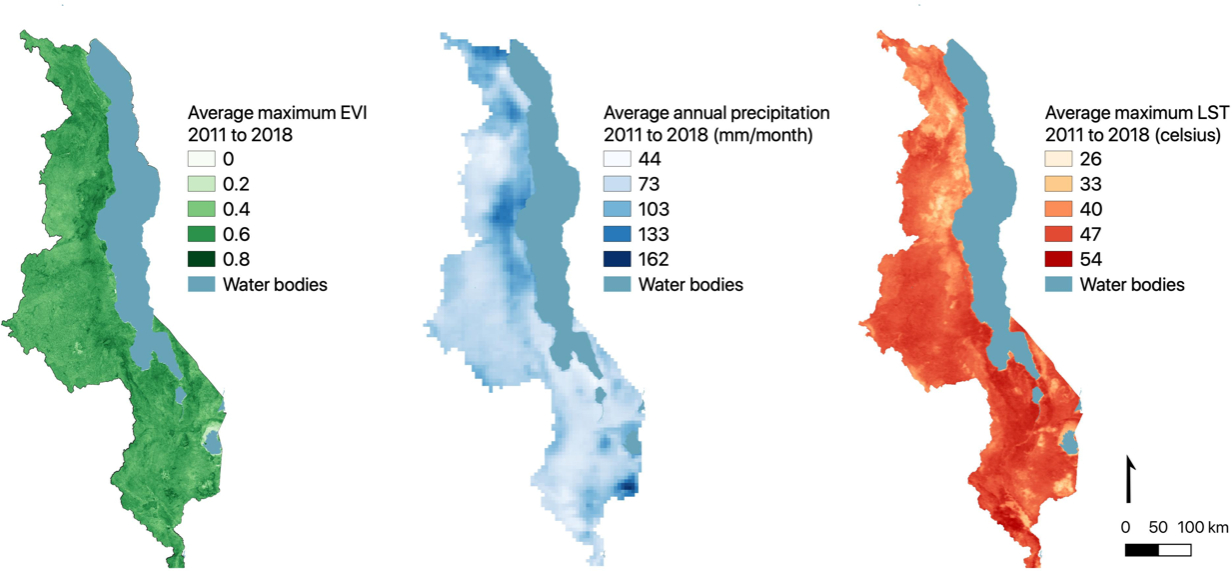

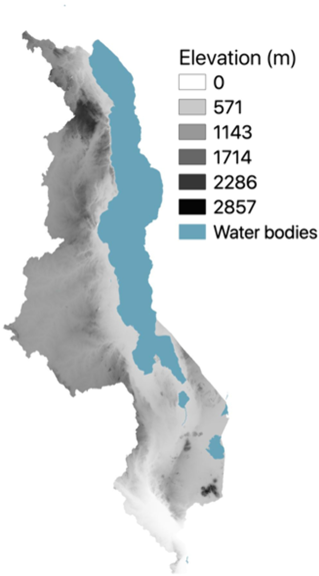


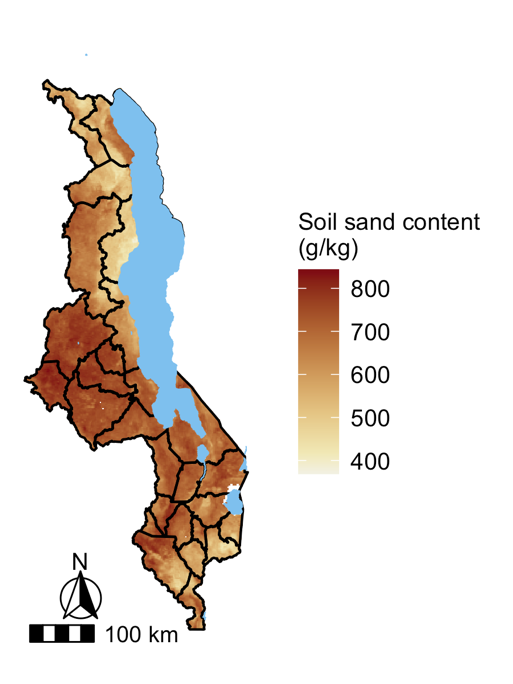

Supplement: S1 Fig — Base map from GADM: https://gadm.org/download_country.html. (DOCX) [file pntd.0012639.s001.docx]

S2 Fig


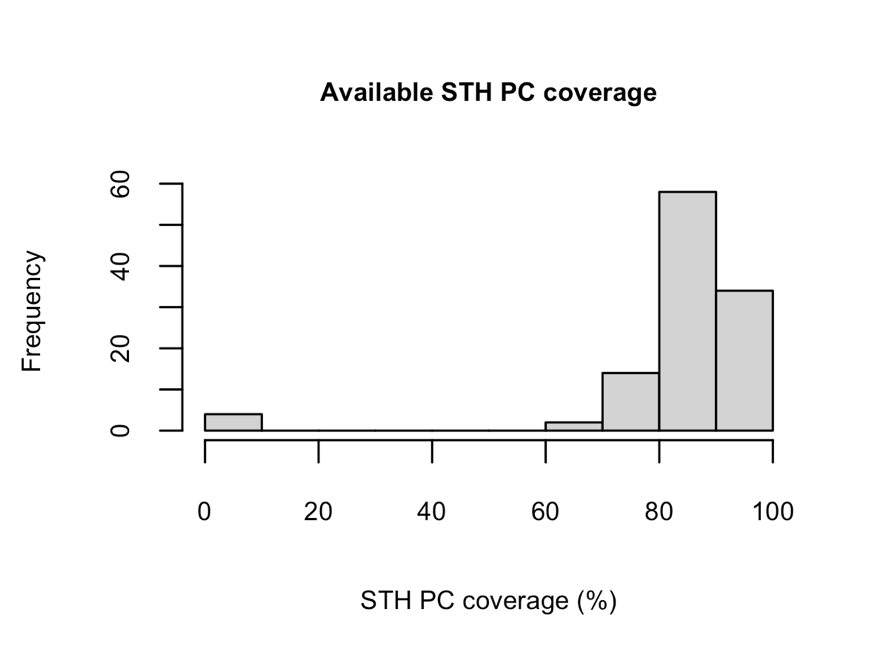

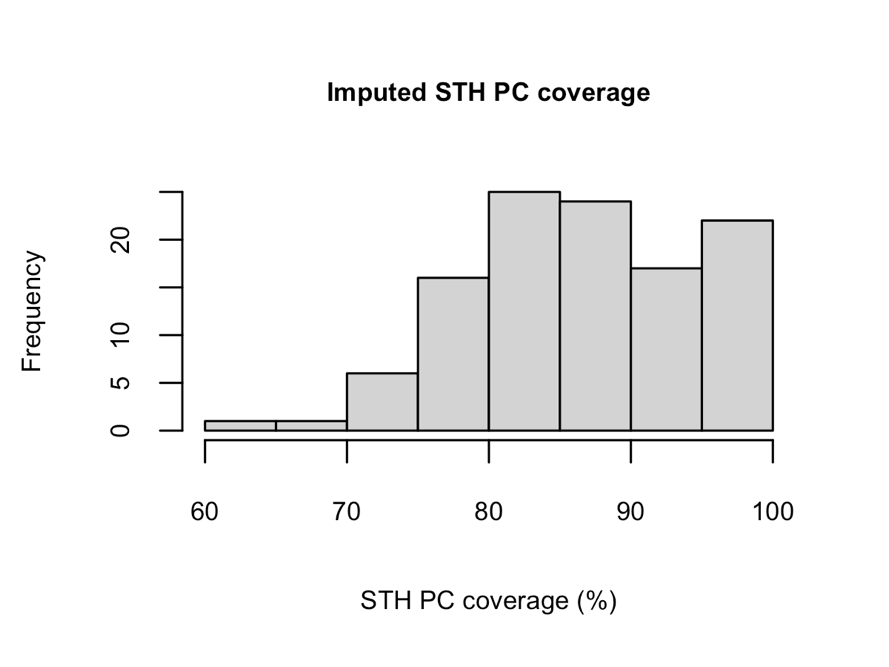

Supplement: S2 Fig — (DOCX) [file pntd.0012639.s002.docx]

S3 Fig


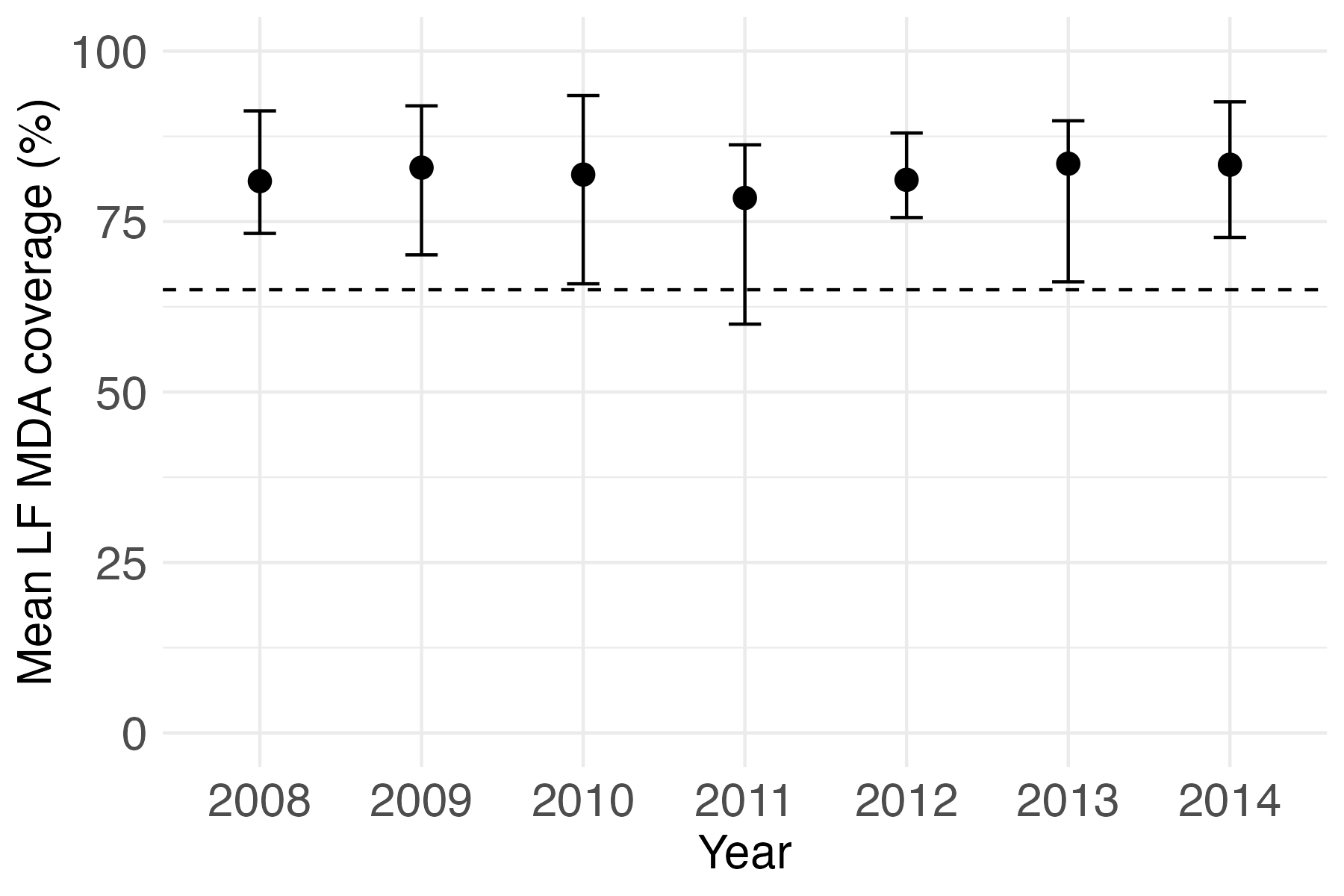

Supplement: S3 Fig — Horizontal dashed line indicates the 65% coverage target set for LF MDA. (DOCX) [file pntd.0012639.s003.docx]

S4 Fig


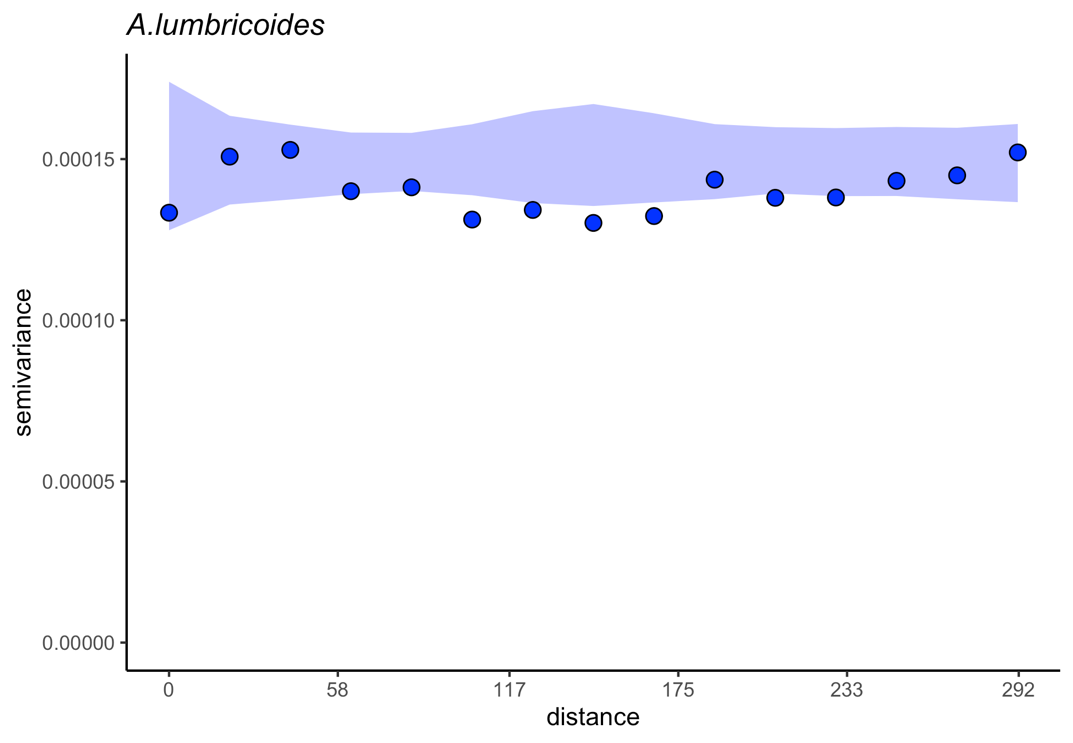

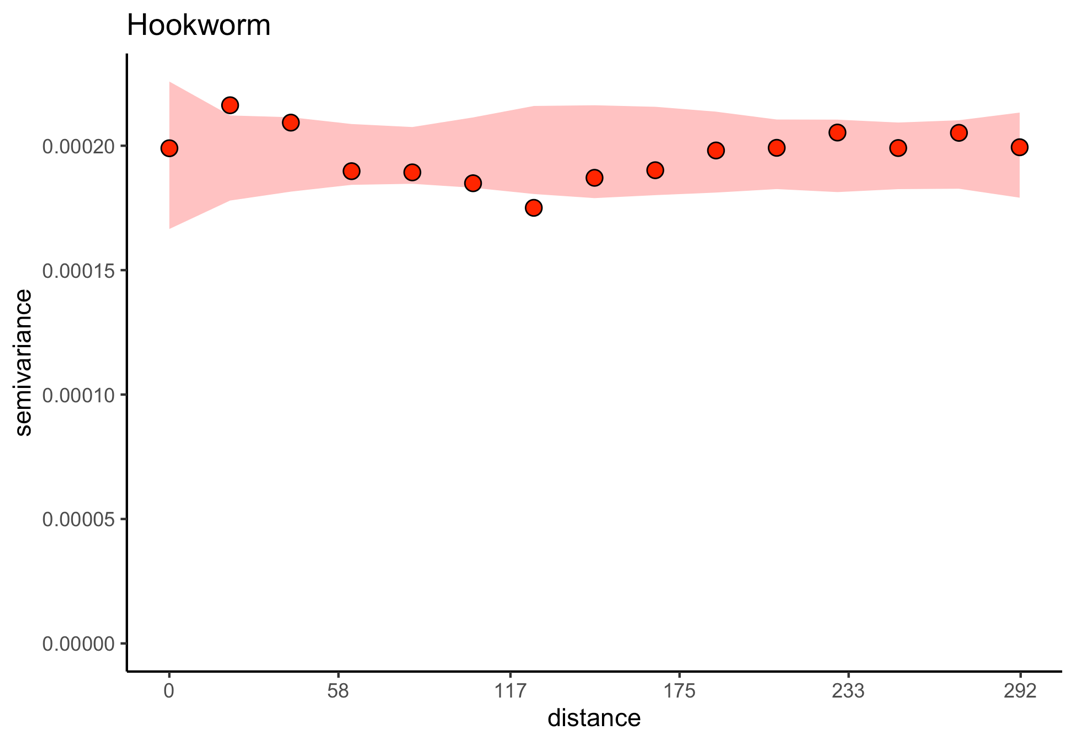

Supplement: S4 Fig — (DOCX) [file pntd.0012639.s004.docx]

S5 Fig


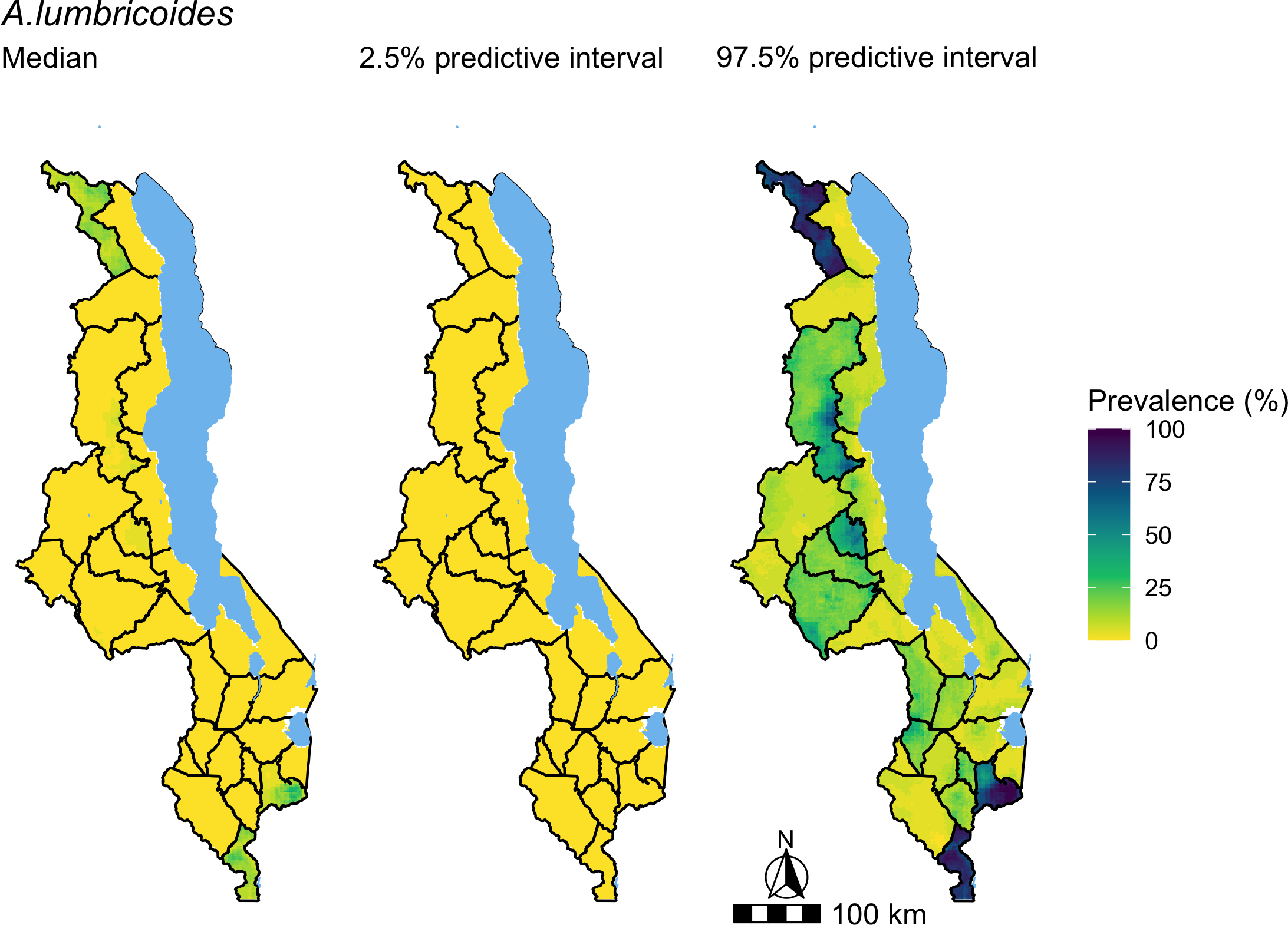


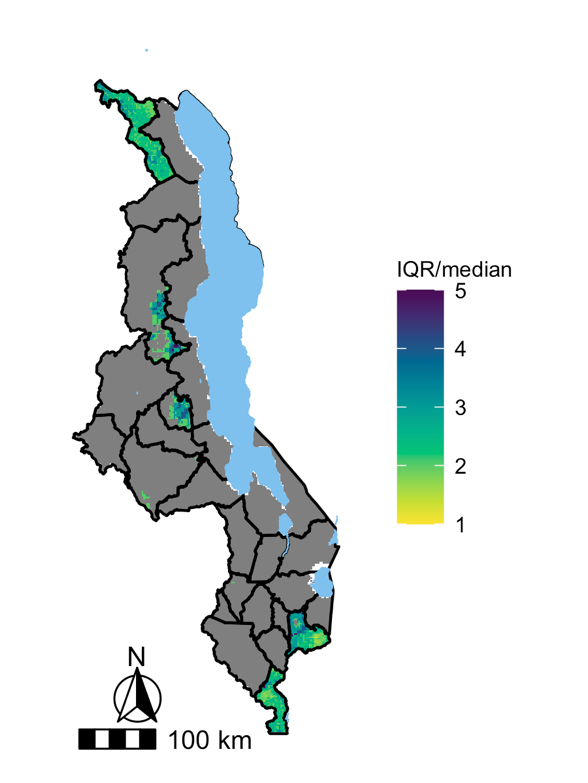

Supplement: S5 Fig — Median of the predicted prevalence after LF MDA alongside 95% predictive intervals and the relative interquartile range (IQR), IQR/median of the predictions where the median is above 0%. Base map from GADM: https://gadm.org/download_country.html. (DOCX) [file pntd.0012639.s005.docx]

S6 Fig


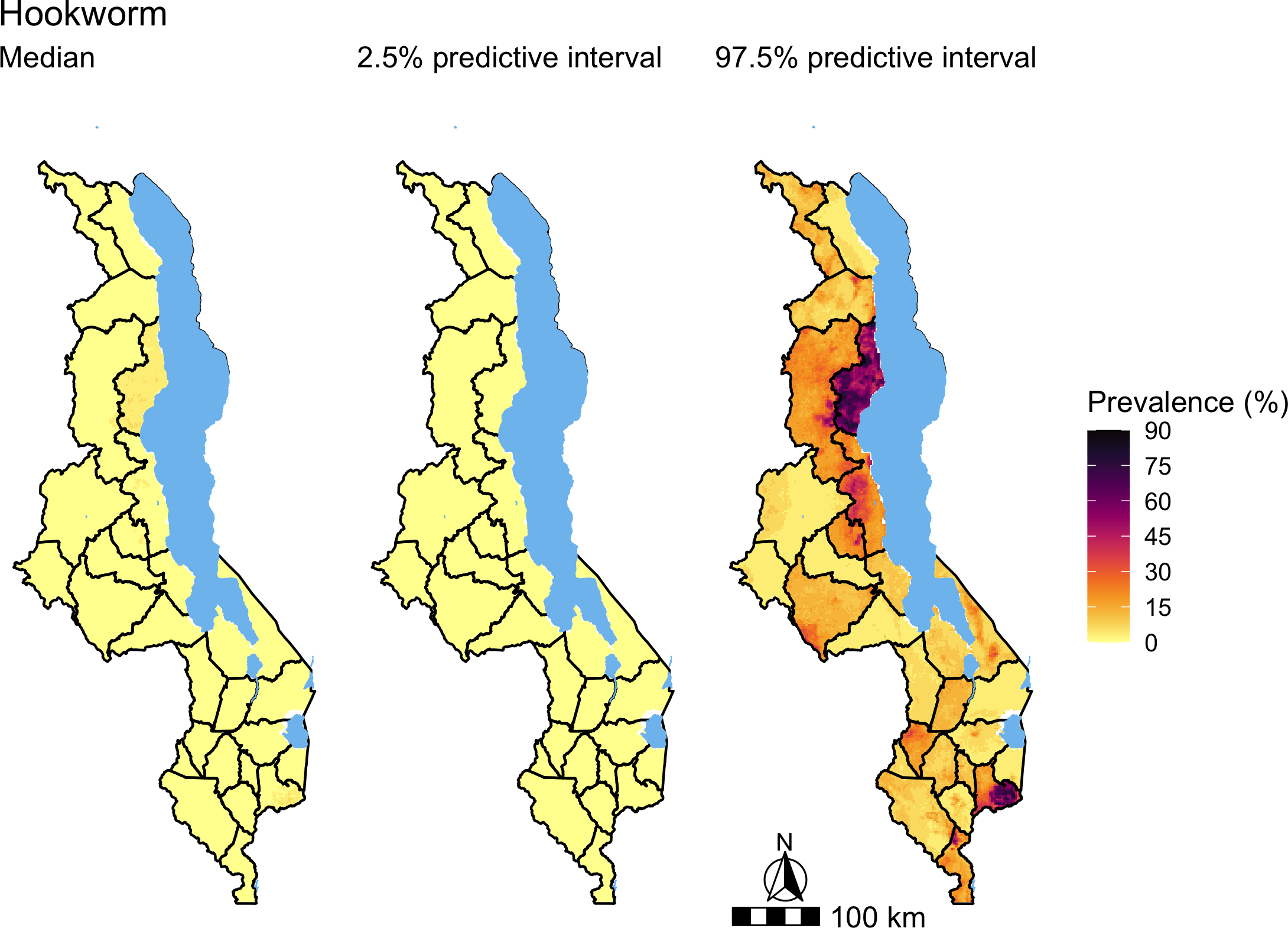

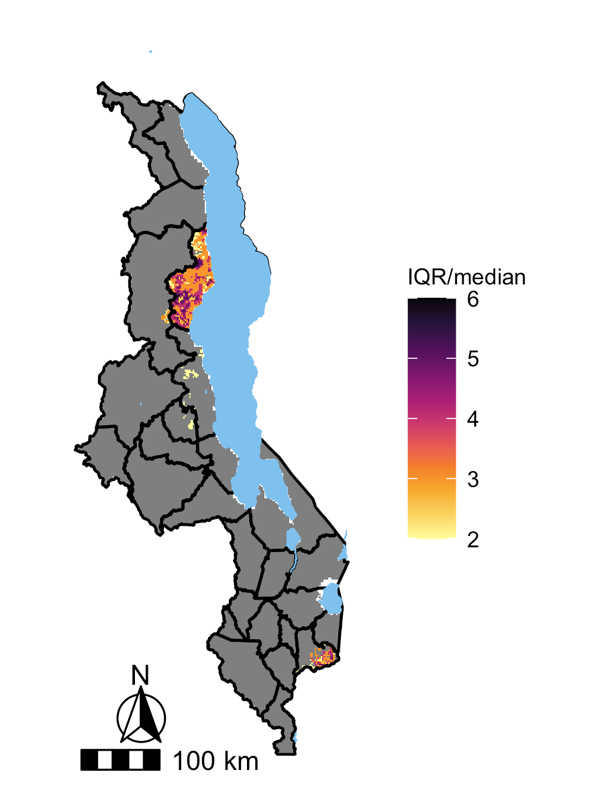

Supplement: S6 Fig — Median of the predicted prevalence after LF MDA alongside 95% predictive intervals and the relative interquartile range (IQR), IQR/median of the predictions where the median is above 0%. Base map from GADM: https://gadm.org/download_country.html. (DOCX) [file pntd.0012639.s006.docx]

S7 Fig


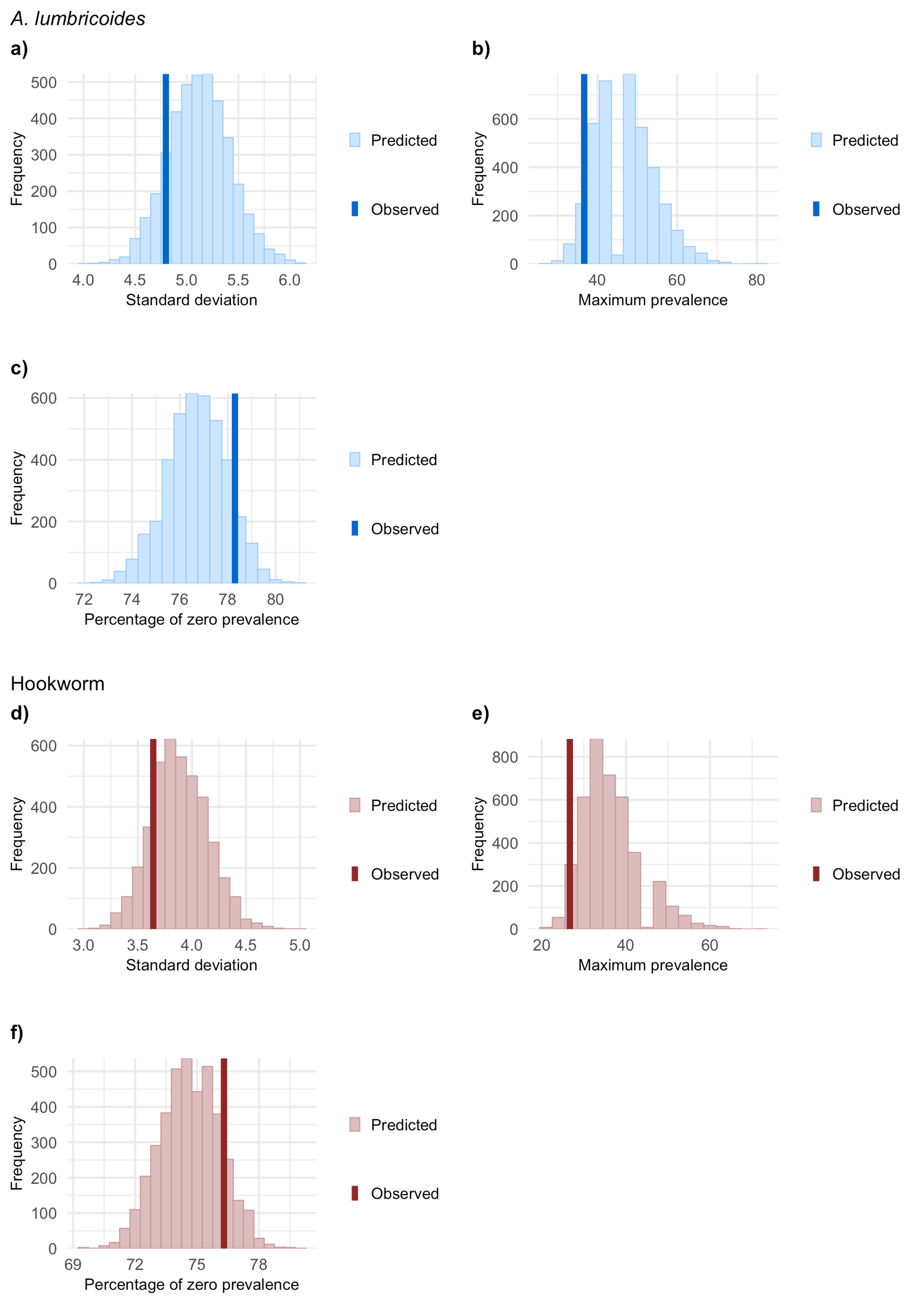

Supplement: S7 Fig — Derived from 4,000 simulations of the predicted prevalence at each school obtained from the full multivariate mixed-effects Bayesian logistic regression models presented in Fig 5. (a-c) The standard deviation, maximum school-level prevalence and percentage of zero prevalence’s predicted by the A. lumbricoides model for each of the 4,000 simulations, compared to its respective observed value in the data. (d-f) The standard deviation, maximum school-level prevalence and percentage of zero prevalence’s predicted by the hookworm model for each of the 4,000 simulations, compared to its respective observed value in the data. (DOCX) [file pntd.0012639.s007.docx]
