# Supplementary material for: The impact of the termination of Lymphatic Filariasis mass drug administration on Soil-transmitted Helminth prevalence in school children in Malawi
Source: PLoS Negl Trop Dis. 2026 Feb 25;20(2):e0012639. doi: 10.1371/journal.pntd.0012639 (PMC12956128; doi:10.1371/journal.pntd.0012639)
Supplement: S1 Table — The change in the variance of the random effects in the A. lumbricoides and hookworm models, with no explanatory variables (empty model) vs with all explanatory variables (full model). (DOCX) [file pntd.0012639.s008.docx]

S1 Table

| ***A. lumbricoides* model** | School random effect variance (95% credible intervals) | District random effect variance (95% credible intervals) |
| --- | --- | --- |
| Empty model | 1.8 (1.56 – 2.02) | 2.0 (1.36 – 2.73) |
| Full model | 1.7 (1.49 – 1.94) | 2.1 (1.38 – 2.94) |
|  |  |  |
| **Hookworm model** | School random effect variance (95% credible intervals) | District random effect variance (95% credible intervals) |
| Empty model | 1.5 (1.30 – 1.70) | 1.5 (0.96 – 2.29) |
| Full model | 1.5 (1.32 – 1.76) | 1.3 (0.75 – 2.10) |
