# Supplementary material for: The impact of the termination of Lymphatic Filariasis mass drug administration on Soil-transmitted Helminth prevalence in school children in Malawi
Source: PLoS Negl Trop Dis. 2026 Feb 25;20(2):e0012639. doi: 10.1371/journal.pntd.0012639 (PMC12956128; doi:10.1371/journal.pntd.0012639)
Supplement: S1 Appendix — (DOCX) [file pntd.0012639.s009.docx]

S1 Appendix

Data sources

| **Variable** | **Source** | **Spatial resolution** | **Temporal resolution** |
| --- | --- | --- | --- |
| Land Surface Temperature (kelvin) | Terra MODIS version 6.0 products (1) | 1km | Eight day average, 2011 to 2019 |
| Enhanced Vegetation Index | Terra MODIS version 6.0 products (2) | 250m | 16 day composite, 2011 to 2019 |
| Precipitation (mm) | Climate Hazards Group InfraRed Precipitation with Station Data (CHIRPS) project (3) | 0.05 degrees | Monthly, 2011 to 2019 |
| Elevation (m) | Shuttle Radar Topography Mission (SRTM) (4) | 30m | NA |
| Fraction of soil sand content, 0-5cm depth | ISRIC World Soil Information (5) | 1km | NA |
| Population density | WorldPop database (6) | 100m | Yearly, 2011 to 2019 |
| Proportion of individuals who lived under $2 a day | WorldPop database (6) | 1km | 2010-2011 |
| The percentage of households with access to unimproved or improved sanitation and unimproved or improved water source | Predictions based on a Bayesian geospatial model previously published (7) | 5km | Yearly, 2011 to 2017 |

Imputation of missing STH PC coverage

First, the probability of positive coverage, i.e. STH PC coverage above 0%, was modelled for each missing value by fitting a logistic regression model with the non-missing STH PC coverage as the outcome and the available variables as inputs, which included the environmental, demographic, WASH and MDA variables, in addition to district and year of coverage. This model was then used to predict whether STH PC coverage exceeded 0%, for each missing value. Secondly, a linear regression model was fitted using the non-missing STH PC coverage that were positive (STH PC coverage > 0%) as the outcome and the same variables as the logistic regression model as inputs to predict the percentage coverage of STH PC for each missing value. Lastly, the missing value was imputed as the product of the predicted outcome from the logistic regression model and the predicted level of coverage from the linear model.

Variograms to assess presence of spatial correlation

The following Bayesian mixed-effects logistic regression model was fitted for each parasite, with their respective final list of variables, using the rstanarm package (8). Weakly informative prior distributions, a normal distribution with mean 0 and variance 2.5, were used for the intercept and the coefficients. A variogram was then created to assess the presence of residual spatial correlation.

$$\log\left( \frac{P\left( x_{i} \right)}{1-P\left( x_{i} \right)} \right)=\alpha+u_{i}+\boldsymbol{d}\left( x_{i} \right)^{T}\boldsymbol{\beta}$$

$$u_{i} \sim N\left( 0, \sigma^{2} \right)$$

Here the log-odds of the infection prevalence of each parasite at school $x_{i}$ is equal to the global intercept$\left( \alpha\right),$ plus the random effect for school $x_{i}$ $\left( u_{i} \right)$ and $\boldsymbol{d}\left( x_{i} \right)^{T}\boldsymbol{\beta}$, the vector of explanatory variables at school $x_{i}$ with $\beta$ as their coefficients, which represent the fixed effects. The random effects $\left( u_{i} \right)$ at each school are a set of independent zero-mean Gaussian variables with variance $\sigma^{2}$. The variogram was then created by plotting the squared differences in the estimates of the random effects at each school location, e.g. difference in $u_{i}$ and $u_{j}$ at schools $x_{i}$ and $x_{j}$, against the distance between the schools. Under the assumption of spatial independence, 95% confidence intervals were created through 10,000 repetitions of the variogram, each time randomly reassigning the estimated random effects to schools (9). The presence of spatial correlation is then indicated if the computed variogram falls outside of the 95% confidence interval limits (9).

Change in variance after inclusion of explanatory variables

The relative change in the school-level and district-level variance were computed using the following formula respectively.

$$\frac{\sigma_{u}^{2} \left( empty \right)- \sigma_{u}^{2}(full)}{\sigma_{u}^{2} \left( empty \right)} \times100$$

(1)

$$\frac{\sigma_{v}^{2} \left( empty \right)- \sigma_{v}^{2}(full)}{\sigma_{v}^{2} \left( empty \right)} \times100$$

(2)

Where the percentage change was calculated using the random effects variance, school-level ($\sigma_{u}^{2}$) or district-level ($\sigma_{v}^{2}$), from the full model (including all explanatory variables) and the empty model (random effects only, no explanatory variables). This was done for both the A. lumbricoides and hookworm models. Any change in the random effects variance was due to the addition of the explanatory variables.

Assessment of model fit

Posterior predictive checks were conducted on the final Bayesian mixed-effects model for *A. lumbricoides* and hookworm infection, to examine the model’s ability to predict prevalence resembling the observed data as an assessment of model fit (10). The predicted prevalence at each school was generated using 4,000 simulations from the model, where each simulation was one set of predictions for all schools in the dataset. Statistical summaries were then created for each of the 4,000 simulations and plotted as a distribution, which was then compared to the observed data. These summaries included the standard deviations of the prevalence predicted by the model, which its distribution was compared to the standard deviation of the observed prevalence. The distribution of maximum prevalence’s predicted by the model was compared to the observed maximum prevalence and the proportion of zero prevalence schools predicted was compared to the proportion of zero prevalence schools in the observed data.

References

1. Wan Z, Hook S, Hulley G. MOD11A2 MODIS/Terra Land Surface Temperature/Emissivity 8-Day L3 Global 1km SIN Grid V006</i> [Data set]. NASA EOSDIS Land Processes DAAC. 2015

2. Didan K. MOD13Q1 MODIS/Terra Vegetation Indices 16-Day L3 Global 250m SIN Grid V006</i> [Data set]. NASA EOSDIS Land Processes DAAC. 2015

3. Funk C, Peterson P, Landsfeld M, Pedreros D, Verdin J, Shukla S, et al. The climate hazards infrared precipitation with stations—a new environmental record for monitoring extremes. Scientific Data. 2015;2(1):150066.

4. EROS. USGS EROS Archive - Digital Elevation - Shuttle Radar Topography Mission (SRTM) 1 Arc-Second Global 2018 [Available from: <https://www.usgs.gov/centers/eros/science/usgs-eros-archive-digital-elevation-shuttle-radar-topography-mission-srtm-1?qt-science_center_objects=0#qt-science_center_objects>.

5. Poggio L, De Sousa LM, Batjes NH, Heuvelink GBM, Kempen B, Ribeiro E, et al. SoilGrids 2.0: producing soil information for the globe with quantified spatial uncertainty. SOIL. 2021;7(1):217–40.

6. WorldPop. 2023 [Available from: <https://www.worldpop.org/>.

7. Deshpande A, Miller-Petrie MK, Lindstedt PA, Baumann MM, Johnson KB, Blacker BF, et al. Mapping geographical inequalities in access to drinking water and sanitation facilities in low-income and middle-income countries, 2000&#x2013;17. Lancet Glob Health. 2020;8(9):e1162–e85.

8. Goodrich B, Gabry J, Ali I, Brilleman S. rstanarm: Bayesian applied regression modeling via Stan. 2023.

9. Diggle PJ, Giorgi E. Model-based Geostatistics for Global Public Health. 1st ed ed: Chapman and Hall/CRC; 2019.

10. Gabry J, Simpson D, Vehtari A, Betancourt M, Gelman A. Visualization in Bayesian workflow. Journal of the Royal Statistical Society: Series A (Statistics in Society). 2019;182(2):389–402.
